# Protective and Regenerative Effects of Reconstituted HDL on Human Rotator Cuff Fibroblasts under Hypoxia: An In Vitro Study

**DOI:** 10.3390/antiox13040497

**Published:** 2024-04-22

**Authors:** Ra Jeong Kim, Hyung Bin Park

**Affiliations:** 1 Institute of Medical Sciences, Gyeongsang National University, Jinju 52727, Republic of Korea; younme1112@gnu.ac.kr; 2 Department of Orthopaedic Surgery, School of Medicine, Gyengsang National University, Jinju 52727, Republic of Korea; 3 Department of Orthopaedic Surgery, Gyengsang National University Changwon Hospital, Changwon 51472, Republic of Korea

**Keywords:** rotator cuff tear, fibroblasts, hypoxia, reconstituted HDL, antioxidants, cell proliferative effects, apoptosis

## Abstract

Hypoxia and hypo-high-density lipoproteinemia (hypo-HDLemia) are proposed risk factors for rotator cuff tear. HDL is recognized for its potential benefits in ischemia-driven angiogenesis and wound healing. Nevertheless, research on the potential benefits of reconstituted HDL (rHDL) on human rotator cuff fibroblasts (RCFs) under hypoxia is limited. This study investigates the cytoprotective and regenerative effects of rHDL, as well as N-acetylcysteine (NAC), vitamin C (Vit C), and HDL on human RCFs under hypoxic conditions. Sixth-passage human RCFs were divided into normoxia, hypoxia, and hypoxia groups pretreated with antioxidants (NAC, Vit C, rHDL, HDL). Hypoxia was induced by 1000 µM CoCl_2_. In the hypoxia group compared to the normoxia group, there were significant increases in hypoxia-inducible factor-1α (HIF-1α), heme oxygenase-1 (HO-1), and Bcl-2/E1B-19kDa interacting protein 3 (BNIP3) expressions, along with reduced cell viability, elevated reactive oxygen species (ROS) production, apoptosis rate, expressions of cleaved caspase-3, cleaved poly ADP-ribose polymerase-1 (PARP-1), vascular endothelial growth factors (VEGF), and matrix metalloproteinase-2 (MMP-2), as well as decreased collagen I and III production, and markedly lower cell proliferative activity (*p* ≤ 0.039). These responses were significantly mitigated by pretreatment with rHDL (*p* ≤ 0.046). This study suggests that rHDL can enhance cell proliferation and collagen I and III production while reducing apoptosis in human RCFs under hypoxic conditions.

## 1. Introduction

Despite the high morbidity of rotator cuff tendinopathy, or tendon degeneration, its causes and involved factors remain incompletely elucidated [1,2]. Hypovascularity has been proposed as a cause of rotator cuff tendon tear, but this remains controversial [3,4,5,6]. Excessive apoptosis has been reported to be associated with rotator cuff tendinopathy [7,8,9]. The supraspinatus tendon, which is the tendon most commonly involved in rotator cuff disease, exhibits, as a gliding tendon, a characteristic associated with hypoxia: a fibrocartilaginous portion close to the insertion site [10]. Hypoxia induces stress in organisms through either physiological or pathological conditions [11]. Recent musculoskeletal research reported hypoxia as associated with tendinopathy occurring in several tendons; that study suggested critical pathophysiological mechanisms of tendinopathy [12]. In vitro studies have shown that cultured fibroblasts undergoing cyclical strain induce hypoxia-inducible factor-1α (HIF-1α) and vascular endothelial growth factors (VEGF), markers related to hypoxia [13]. Based on the findings of increased expression of hypoxic markers of HIF-1α and Bcl-2/E1B-19kDa interacting protein 3 (BNIP3), as well as increased rates of apoptosis in impingements and torn rotator cuff tendons, one study proposed that hypoxic changes in rotator cuff tendons contribute to the development of rotator cuff tear [14]. From a molecular biological perspective, hypoxia induces HIF-1α, which in turn triggers the expression of VEGF. Subsequently, VEGF stimulates endothelial cells and vessels to invade hypovascularized tissue [15]. Some studies have found higher concentrations of VEGF in degenerative Achilles tendons than in healthy tendon tissues [10,16]. There is some evidence that neovascularization is also a factor in rotator cuff tendinopathy [5,17]. Increased HIF and VEGF expressions in torn rotator cuff tendons have been reported [8]. These findings support the involvement of hypoxia in rotator cuff tendon degeneration.

Dyslipidemia and its subtype, hypo-high-density lipoproteinemia (hypo-HDLemia), have been reported as risk factors for both rotator cuff tear and retear after repair [1,18,19]. High-density lipoprotein (HDL), commonly known as the ‘good cholesterol’, plays a major role in regulating cholesterol metabolism [20]. HDL also exhibits antioxidative, antithrombotic, and anti-inflammatory properties [21]. Several studies suggest that HDL is positively associated with ischemia-driven angiogenesis, a process essential for wound healing under hypoxic conditions [20,22,23,24]. Recent studies have reported that HDL augments angiogenesis under conditions of hypoxia; those studies suggest a possible role in therapeutic modulation of ischemic injury [25,26]. For tenocytes at risk of hypoxic death, pro-survival growth factors including insulin-like growth factor and platelet-rich plasma, as well as N-acetylcysteine (NAC), an antioxidant, have been proposed as potentially protective [27,28]. Reconstituted HDL (rHDL) is produced in a laboratory setting by combining purified apolipoprotein A-I, the main protein component of HDL, with phospholipids, the main lipid component of HDL [29,30]. rHDL is being experimentally used for treating cardiovascular diseases and delivering drugs [29,30]. Despite recent advances in rHDL research, there is currently limited knowledge of the cytoprotective and wound-healing effects of rHDL on rotator cuff fibroblasts (RCFs) under hypoxia. Therefore, this study aimed to investigate whether rHDL can exert such effects on human RCFs under hypoxia.

## 2. Materials and Methods

### 2.1. Clinical Sample Collection

Primary RCFs were obtained from patients who underwent arthroscopic rotator cuff repair with approval from the Institutional Review Board of Gyeongsang National University (GNU-170918-R0043). The tissues were washed twice with PBS (Lonza, Walkersville, MD, USA), minced into small pieces with a sterile scalpel, and cultured on a 6-well tissue culture plate (Thermo Fisher Scientific, Waltham, MA, USA) in DMEM (Thermo Fisher Scientific, Waltham, MA, USA) supplemented with 20% FBS (Thermo Fisher Scientific, Waltham, MA, USA) and 1% Antibiotic-Antimycotic (Thermo Fisher Scientific, Waltham, MA, USA) in a humidified 5% CO_2_ atmosphere at 37 °C. After two weeks, the cells had reached 90% confluence. The cells were then trypsinized using TrypLE^TM^ Express (Thermo Fisher Scientific, Waltham, MA, USA) and 0.02% EDTA in PBS (Lonza, Walkersville, MD, USA) for 5 min, centrifuged at 1300 rpm for 3 min, and expanded in a second passage. The cells were harvested using TrypLE^TM^ Express (Thermo Fisher Scientific, Waltham, MA, USA), cryopreserved, and later thawed for use in the study at the 3–6 passage. The cultured cells were characterized as tendon fibroblasts using markers specific to fibroblasts and tendon cells (Supplement Figure S1).

### 2.2. Reagents

This study used reagents including CoCl_2_ (Sigma-Aldrich, St. Louis, MO, USA), rHDL (Avivasystem bio, San Diego, CA, USA), a complex of human Apolipoprotein A-I and 1-palmitoyl-2-oleoyl phosphatidylcholine (at a molar ratio of 1 to 100), as well as HDL, vitamin C (Vit C), and NAC (all from Sigma-Aldrich, St. Louis, MO, USA).

### 2.3. Study Design

This study has been approved by the Institutional Review Board of the Gyeongsang National University (IRB: GNU-170918-R0043). The human RCFs were divided into the study groups of normoxia, hypoxia, NAC-hypoxia, Vit C-hypoxia, rHDL-hypoxia, and HDL-hypoxia. Hypoxia was induced using 1000 µM CoCl_2_, a well-known chemical hypoxic agent [31,32], based on a preliminary study. This study evaluated the expressions of HIF-1α, heme oxygenase-1 (HO-1), and BNIP3, rates of cell viability, intracellular reactive oxygen species (ROS) production, and apoptosis, as well as the expressions of cleaved caspase-3, cleaved poly ADP-ribose polymerase-1 (PARP-1), vascular endothelial growth factors (VEGF), matrix metalloproteinase-2 (MMP-2), collagen I and III production, and cell proliferative ability (Supplement Figures S2–S7). The cells were exposed to CoCl_2_ for 24 h, except for HIF-1α (4 h), VEGF (1 h), and ROS (1 min). Antioxidants—10 mM NAC, 2 mM Vit C, 200 µM rHDL, and 200 µM HDL—were treated for 1 h before exposure to CoCl_2_. The concentration of antioxidants was chosen according to the 2,2-diphenyl-1-picrylhydrazyl (DPPH) radical scavenging activity (Supplement Figure S8).

### 2.4. Measurement of Intracellular ROS Production

The intracellular ROS production of each study subset was qualitatively assessed using a fluorescence microscope. Human RCFs with 1 × 10^4^ RCFs were seeded in a dish and incubated for 24 h. After washing with PBS and the addition of serum-free medium, the cells were incubated with a 5 μmol/L DCF-DA solution for 15 min at 37 °C. Finally, the intracellular ROS production was analyzed using a fluorescence microscope (Nikon, Ti2-U FL, Tokyo, Japan).

### 2.5. Western Blot Analyses

After exposing human RCFs (3 × 10^5^) to various studied reagents according to the study’s subsets, the studied cells were washed with cold PBS, and total cell lysates were prepared by scraping in 100 µL of RIPA buffer (Thermo Fisher Scientific, Waltham, MA, USA). The digested cells were then sonicated and centrifuged at 13,000 rpm for 20 min at 4 °C to remove insoluble debris. The samples were resolved on 8~12% SDS-polyacrylamide gel and then electrophoretically transferred onto a PVDF membrane using the wet technique. The membrane was then blocked for 1 h with 5% skim milk in a TBS-T buffer solution (IBS-BT008, iNtRon, Seongnam, Republic of Korea) and incubated with primary antibodies against HIF-1α (1: 10,000, A300-286A, BETHYL Laboratories, Montgomery, TX, USA), HO-1, cleaved caspase-3, cleaved PARP-1, MMP-2, (1: 1000, #43966, #9662, #9542, #40994, Cell Signaling Technology, Danvers, MA, USA), BNIP3, Collagen I, Collagen III (1:1000, ab1093, ab138492, ab184993, abcam, Cambridge, UK), VEGF (1: 100, sc-7269, Santa Cruz, CA, USA), and β-actin (1: 10,000, MA1-744, Thermo Fisher Scientific, Waltham, MA, USA) in TBS-T buffer containing 5% skim milk (Biopure, Seoul, Republic of Korea). Specific antibody binding was detected by horseradish peroxidase-conjugated secondary antibodies (anti-rabbit and anti-mouse, 1: 5000; 1460, 31430, Thermo Fisher Scientific, Waltham, MA, USA) and visualized using an enhanced chemiluminescence detection reagent (Thermo Fisher Scientific, Waltham, MA, USA).

### 2.6. Cell Viability Analyses

The MTT assay (Sigma-Aldrich, St. Louis, MO, USA) and LIVE/DEAD Viability/Cytotoxicity Kit (Invitrogen, Carlsbad, CA, USA) were used to evaluate cell viability.

For the MTT assay, human RCFs (2 × 10^4^) were seeded in a 24-well plate and exposed to different agents for 24 h before adding MTT solution. Absorbance was measured at 570 nm to determine cell viability. Cell viability was evaluated by using the MTT. Human RCFs (2 × 10^4^) were seeded in each well of a 24-well plate. The cells were maintained in an incubator at 5% CO_2_, 37 °C for 24 h. Each study group was exposed to culture media, NAC, Vit C, rHDL or HDL, and CoCl_2_ according to the study subset. A 500 μL MTT solution (0.5 mg/mL in free media) was briefly added to each well of the 24-well plate. Then, the plate was incubated for 2 h. Afterwards, the cell supernatant was removed and 200 μL DMSO (Merck, Darmstadt, Germany) was added to each well of the plate. The absorbance of the plate was measured at 570 nm, using a microplate reader. Cell viability was expressed as a percentage of live cells, compared with the control, which was set at 100%.

Cell viability was also assessed using the LIVE/DEAD Viability/Cytotoxicity Kit (Invitrogen, Carlsbad, CA, USA). Human RCFs (1 × 10^5^) were seeded in a 35mm confocal dish. The cells were maintained in an incubator at 5% CO_2_, 37 °C for 24 h. The cells were treated with various studied agents according to the study subset. Briefly, a Live/Dead kit solution (5× dye) was added to the dish. After the dish was incubated for 10 min at room temperature, the cells were evaluated using a fluorescence microscope (Nikon, Ti2-U FL, Tokyo, Japan); digital photographs were taken at 100 magnifications.

### 2.7. Analyses for Apoptosis Rates

FACS analyses were performed for the analyses of apoptosis rates using Annexin V/PI double staining. Human RCFs (1 × 10^5^) were seeded in each well of a 6-well plate. After 24 h incubation, the study groups were exposed to various studied drugs according to their study subset. Human RCFs were harvested after trypsinization, then centrifuged and collected. Those cells were washed with PBS and then stained using an FITC Annexin V/PI kit (BD Biosciences, Franklin Lakes, NJ, USA), according to the manufacturer’s instructions. Using flow cytometry (Cytomics FC500, Beckman Coulter, Brea, CA, USA), cell viability was determined as follows: live cells were labeled with neither stain; early apoptotic cells were labeled only with Annexin V; necrotic cells were labeled only with PI; and apoptotic cells were labeled with both Annexin V and PI.

TUNEL assay was performed to detect apoptotic cells, using a TUNEL kit (Roche Applied Science, Indianapolis, IN, USA), according to the manufacturer’s protocol. Briefly, Human RCFs (1 × 10^4^) were seeded in a confocal dish. After 24 h incubation, the study groups were exposed to various studied drugs, according to each study’s subset. The cells were counterstained with DAPI (4′,6-diamidino-2-phenylindole, Sigma-Aldrich, St. Louis, MO, USA). The cells were then evaluated at 200× magnification using a fluorescence microscope (Nikon, Ti2-U FL, Tokyo, Japan). The percentage of apoptotic cells was calculated as the ratio of TUNEL-positive cells to DAPI-stained cells.

### 2.8. Analyses of Cellular Proliferation

Cellular proliferation was assessed using Ki-67 staining. Human RCFs (1 × 10^5^) were seeded in a 35mm confocal dish. After 24 h incubation, the study groups were exposed to various studied agents, according to each study’s subset. A fixative solution of cold methanol was added to each well, which was then incubated for 20 min at 4 °C. The cells were permeabilized with 0.3% Triton X-100 (Sigma-Aldrich, St. Louis, MO, USA) added to each well, which was then incubated for 20 min at room temperature. The cells were then incubated in 1% bovine serum albumin (Amresco, Solon, OH, USA) in PBS for 1 h at room temperature. After that, the 1:200 diluted Ki-67 (ab15580, Abcam, Cambridge, MA, USA) and β-actin (Thermo Fisher Scientific, Waltham, MA, USA) primary antibody was added, and the cells were incubated for 2 h at room temperature. The secondary antibodies (ab150119, Goat Anti-Mouse IgG H&L Alexa Fluor^®^ 647/ab150081, Goat-Anti-Rabbit IgG H&L Alexa Fluor^®^ 488, abcam, Cambridge, MA, USA) were used at the 1:200 dilutions for 1 h at room temperature and cells were counterstained with 1 μg/mL of DAPI (4′,6-diamidino-2-phenylindole, Thermo Fisher Scientific, Waltham, MA, USA). The cells were then evaluated through a fluorescence microscope (Nikon, Ti2-U FL, Tokyo, Japan); digital photographs were taken at 200 magnifications.

### 2.9. Statistical Analysis

Data were represented as mean ± standard deviation (SD). The one-way ANOVA compared mean levels of the explored parameters, followed by multiple comparisons using Tukey’s method. Statistical significance was set at *p* < 0.05, indicating statistically significant differences. All statistical analyses were performed using GraphPad Prism 9.0 (GraphPad Software Inc., San Diego, CA, USA).

## 3. Results

### 3.1. Rates of Intracellular ROS Production

**Figure 1:**
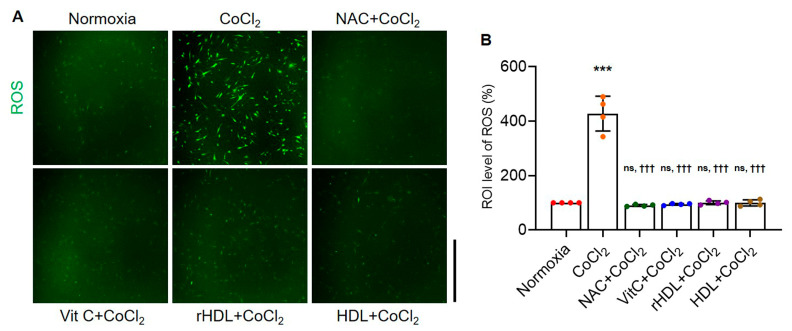
rHDL suppresses CoCl_2_-induced intracellular reactive oxygen species (ROS) production. (**A**,**B**) Fluorescence microscope analyses show that reconstituted HDL (rHDL), as well as other antioxidants, significantly suppresses CoCl_2_-induced intracellular ROS production (*p* < 0.001). Magnification: 4×. Scale bar: 2500 μm. Data are shown as mean ± SD (n = 4). One data point represents the mean of technical replicates of an independent experiment. ***: *p* < 0.001 is compared with normoxia. ^†††^: *p* < 0.001 is compared with the hypoxia group. ^ns^: denotes not significantly different from normoxia.

Intracellular ROS production rates were significantly higher in the hypoxia group than in the normoxia group (*p* < 0.001), while the hypoxia group pretreatment with NAC, Vit C, rHDL, or HDL significantly lowered intracellular ROS production rates compared to the hypoxia group (*p* < 0.001) (Figure 1).

### 3.2. Analyses of the Expressions of HIF-1α, HO-1, and BNIP3

**Figure 2:**
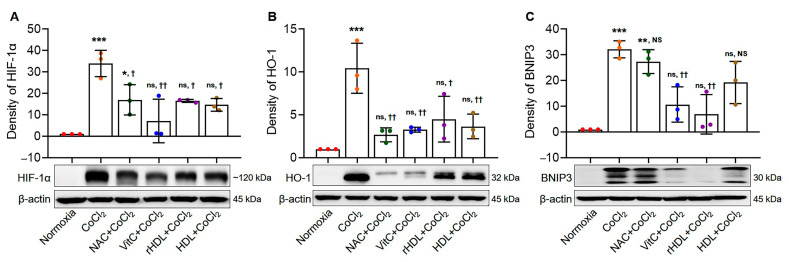
Suppressive effects of rHDL on CoCl_2_-induced hypoxia-inducible factor-1α (HIF-1α), heme oxygenase-1 (HO-1), and Bcl-2/E1B-19kDa interacting protein 3 (BNIP3) expressions. (**A**) HIF-1α expression significantly increased in the hypoxia group compared to the normoxia group (*p* < 0.001). The hypoxia group pretreated with rHDL, as well as the hypoxia groups with N-acetylcysteine (NAC), vitamin C (Vit C), and high-density lipoprotein (HDL), exhibit significantly lower HIF-1α expression than the hypoxia group (*p* ≤ 0.034). (**B**) HO-1 expression significantly increased in the hypoxia group compared to the normoxia group (*p* < 0.001). The hypoxia group pretreated with rHDL, as well as the hypoxia groups pretreated with NAC, Vit C, and HDL, exhibit significantly lower HO-1 expression than in the hypoxia group (*p* ≤ 0.013). (**C**) BNIP3 expression significantly increased in the hypoxia group compared to the normoxia group (*p* < 0.001). BNIP3 expressions in the hypoxia group pretreated with rHDL and HDL are significantly lower than in the hypoxia group (*p* ≤ 0.007). Data are shown as mean ± SD (n = 3). *: *p* < 0.05, **: *p* < 0.01, and ***: *p* < 0.001 are compared with normoxia. ^†^: *p* < 0.05 and ^††^: *p* < 0.01 are compared with the hypoxia group. ^ns^: denotes not significantly different from normoxia. ^NS^: denotes not significantly different from the hypoxia group.

The expressions of HIF-1α, HO-1, and BNIP3 were significantly higher in the hypoxia group than in the normoxia group (*p* < 0.001). In the hypoxia groups pretreated with rHDL, as well as NAC, Vit C, or HDL, the expressions of HIF-1α were significantly lower (*p* ≤ 0.034). Similarly, the expressions of HO-1 were significantly lower in the hypoxia groups pretreated with rHDL, as well as NAC, Vit C, or HDL (*p* ≤ 0.013), and the expressions of BNIP3 were significantly lower in the hypoxia groups pretreated with rHDL and HDL compared to the hypoxia group (*p* ≤ 0.007) (Figure 2A–C).

### 3.3. Cell viability Analyses

**Figure 3:**
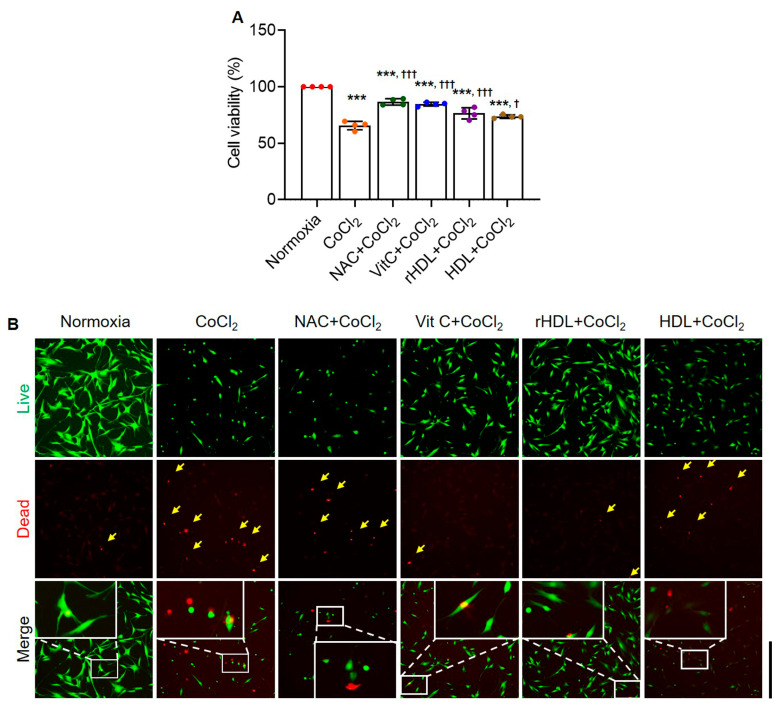
Suppressive effects of rHDL on CoCl_2_-induced cytotoxicity. (**A**) Cell viability in the hypoxia group pretreated with rHDL, as well as the hypoxia groups pretreated with NAC, Vit C, and HDL, was significantly higher than in the hypoxia group (*p* ≤ 0.013). (**B**) Live and dead assay results indicate a markedly higher rate of live cells and a significantly lower rate of dead cells (red) in the hypoxia group pretreated with rHDL, as well as the hypoxia groups pretreated with NAC, Vit C, or HDL, compared to the hypoxia group. The arrow points to the dead cell colored in red. Magnification: 10×, Scale bar: 500 μm, data are shown as mean ± SD (n = 4). One data point represents the mean of technical replicates of an independent experiment. ***: *p* < 0.001 is compared with normoxia. ^†^: *p* < 0.05 and ^†††^: *p* < 0.001 are compared with CoCl_2_.

Cell viability was significantly lower in the hypoxia group than in the normoxia group (*p* < 0.001). Cell viability was significantly higher in the hypoxia groups pretreated with NAC, Vit C, rHDL, or HDL than in the hypoxia group (*p* ≤ 0.013) (Figure 3A). Live and dead assays also indicated that the numbers of dead cells (red) were markedly higher in the hypoxia group than in the normoxia group. Dead cell numbers were markedly lower in the hypoxia groups pretreated with NAC, Vit C, rHDL, or HDL than in the hypoxia group (Figure 3B).

### 3.4. Analyses for Apoptosis

**Figure 4:**
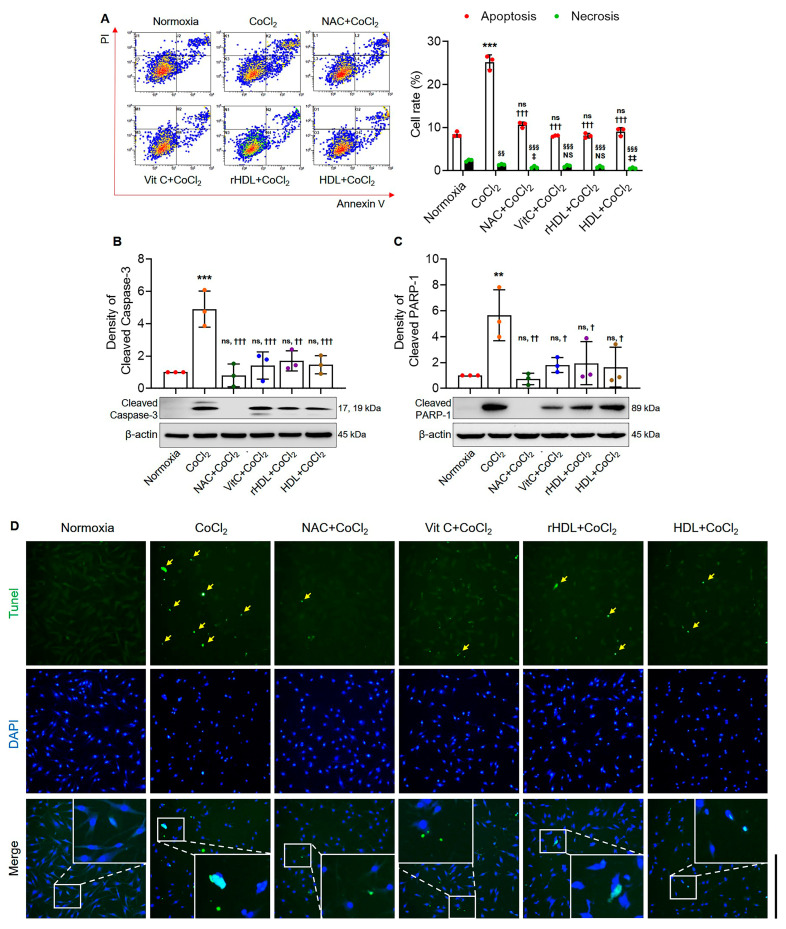
Suppressive effects of rHDL on CoCl_2_-induced apoptosis. (**A**) FACS analyses using annexin V/PI double staining indicate a significantly higher apoptosis rate in the hypoxia group compared to the normoxia group (*p* < 0.001). The apoptosis rate in the hypoxia group pretreated with rHDL, as well as the hypoxia groups pretreated with NAC, Vit C, and HDL, is significantly lower than in the hypoxia group (*p* < 0.001). (**B**) Expression of cleaved caspase-3 is significantly higher in the hypoxia group than in the normoxia group (*p* < 0.001). The expression of cleaved caspase-3 is significantly lower in the hypoxia group pretreated with rHDL, as well as the hypoxia groups pretreated with NAC, Vit C, and HDL, compared to the hypoxia group (*p* ≤ 0.002). (**C**) Expression of cleaved PARP-1 is significantly higher in the hypoxia group than in the normoxia group (*p* = 0.007). The expression of cleaved PARP-1 is significantly lower in the hypoxia group pretreated with rHDL, as well as the hypoxia groups pretreated with NAC, Vit C, and HDL, compared to the hypoxia group (*p* ≤ 0.034). (**D**) TUNEL assay results indicate that apoptotic nuclei (green) are markedly higher in the hypoxia group than in the normoxia group. Apoptotic nuclei are markedly lower in the hypoxia group pretreated with rHDL, as well as the hypoxia groups pretreated with NAC, Vit C, and HDL, compared to the hypoxia group. The arrow points to TUNEL-FITC-positive cells colored in green. Data are shown as mean ± SD (n = 3). Magnification: 10×, Scale bar: 500 μm. **: *p* < 0.01, and ***: *p* < 0.001 are compared with normoxia. ^†^: *p* < 0.05, ^††^: *p* < 0.01, and ^†††^: *p* < 0.001 are compared with the hypoxia group, ^§§^: *p* < 0.01 and ^§§§^: *p* < 0.001 are compared with normoxia, ^‡^: *p* < 0.05 and ^‡‡^: *p* < 0.01 are compared with hypoxia group. ^ns^: denotes not significantly different from normoxia. ^NS^: denotes not significantly different from the hypoxia group.

The apoptosis rate was significantly higher in the hypoxia group than in the normoxia group (*p* < 0.001). The apoptosis rate was significantly lower in the hypoxia groups pretreated with NAC, Vit C, rHDL, or HDL than in the hypoxia group (*p* < 0.001) (Figure 4A). The expression of cleaved caspase-3 was significantly higher in the hypoxia group than in the normoxia group (*p* < 0.001). However, in the hypoxia groups pretreated with NAC, Vit C, rHDL, or HDL, cleaved caspase-3 expression was significantly lower compared to the hypoxia group (*p* ≤ 0.002) (Figure 4B). The expression of cleaved PARP-1 was significantly higher in the hypoxia group than in the normoxia group (*p* = 0.007). However, in the hypoxia groups pretreated with the NAC, Vit C, rHDL, or HDL, cleaved PARP-1 expression was significantly lower compared to in the hypoxia group (*p* ≤ 0.034) (Figure 4C). In the TUNEL assay, apoptotic nuclei (green) were markedly higher in the hypoxia group than in the normoxia group, whereas they were markedly lower in the hypoxia groups pretreated with NAC, Vit C, rHDL, or HDL compared to the hypoxia group (Figure 4D).

### 3.5. Analysis of VEGF and MMP-2 Expressions

**Figure 5:**
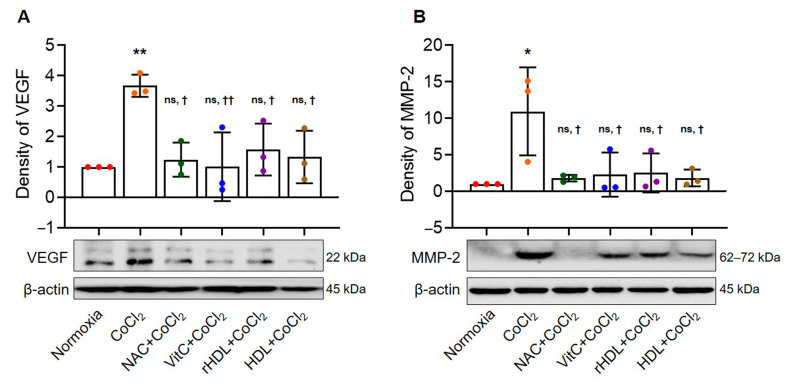
The effects of rHDL against CoCl_2_-induced vascular endothelial growth factors (VEGF) and matrix metalloproteinase-2 (MMP-2). (**A**) Expression of VEGF is significantly higher in the hypoxia group than in the normoxia group (*p* = 0.008). The expression of VEGF is significantly lower in the hypoxia group pretreated with rHDL, as well as the hypoxia groups pretreated with NAC, Vit C, and HDL, compared to the hypoxia group (*p* ≤ 0.038). (**B**) Expression of MMP-2 is significantly higher in the hypoxia group than in the normoxia group (*p* = 0.015). The expression of MMP-2 is significantly lower in the hypoxia group pretreated with rHDL, as well as the hypoxia groups pretreated with NAC, Vit C, and HDL, compared to the hypoxia group (*p* ≤ 0.044). Data are shown as mean ± SD (n = 3). *: *p* < 0.05 and **: *p* < 0.01 are compared with normoxia. ^†^: *p* <0.05 and ^††^: *p* < 0.01 are compared with the hypoxia group. ^ns^: denotes not significantly different from normoxia.

The expression of VEGF was significantly higher in the hypoxia group than in the normoxia group (*p* = 0.008). Expression of VEGF was significantly lower in the hypoxia groups pretreated with NAC, Vit C, rHDL, or HDL than in the hypoxia group (*p* ≤ 0.038) (Figure 5A). The expression of MMP-2 was significantly higher in the hypoxia group than in the normoxia group (*p* = 0.015). However, the expression of MMP-2 was significantly lower in the hypoxia groups pretreated with NAC, Vit C, rHDL, or HDL than in the hypoxia group (*p* ≤ 0.044) (Figure 5B).

### 3.6. Analyses of Collagen I and III Production

**Figure 6:**
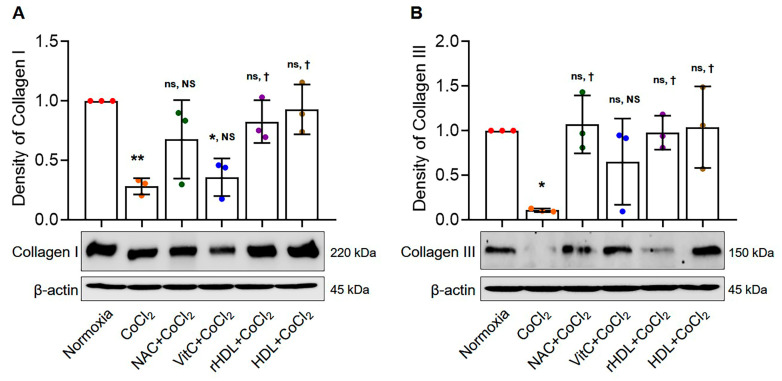
The effects of rHDL against CoCl_2_-induced Collagen I and III. (**A**) Expression of Collagen I is significantly lower in the hypoxia group than in the normoxia group (*p* = 0.006). The expression of Collagen I is significantly higher in the hypoxia group pretreated with rHDL, as well as the hypoxia groups pretreated with HDL, compared to the hypoxia group (*p* ≤ 0.038). (**B**) Expression of Collagen III is significantly lower in the hypoxia group than in the normoxia group (*p* = 0.039). The expression of Collagen III is significantly higher in the hypoxia group pretreated with rHDL, as well as the hypoxia groups pretreated with NAC, and HDL, compared to the hypoxia group (*p* ≤ 0.046). Data are shown as mean ± SD (n = 3). *: *p* < 0.05 and **: *p* < 0.01 are compared with normoxia, ^†^: *p* < 0.05 is compared with the hypoxia group. ^ns^: denotes not significantly different from normoxia. ^NS^: denotes not significantly different from the hypoxia group.

Type I collagen production was significantly lower in the hypoxia group than in the normoxia group (*p* = 0.006). Additionally, type I collagen production was significantly higher in the hypoxia group pretreated with rHDL, as well as the hypoxia groups pretreated with HDL, compared to the hypoxia group (*p* ≤ 0.038) (Figure 6A). Type III collagen production was also significantly lower in the hypoxia group than in the normoxia group (*p* = 0.039). Type III collagen production was significantly higher in the hypoxia group pretreated with rHDL, as well as with NAC or HDL, compared to the hypoxia group (*p ≤* 0.046) (Figure 6B).

### 3.7. Analyses of Cell Proliferation Abilities

**Figure 7:**
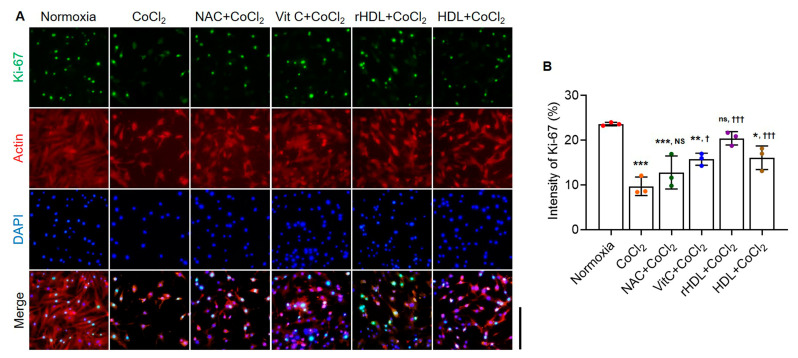
The cell proliferation effects of rHDL against CoCl_2_-induced hypoxia. (**A**,**B**) Ki-67 staining analyses show the cell proliferation effects of studied antioxidants, including rHDL, against CoCl_2_-induced hypoxia. Ki-67 is represented in green, actin in red, and DAPI in blue, with an overlay of both staining. The hypoxia group exhibits a significant decrease in the number of Ki-67 positive cells, indicative of reduced cellular proliferation. Conversely, rHDL, as well as other antioxidant-pretreated groups, shows a significant increase in Ki-67-positive cells, suggesting a cell-proliferative effect. Data are shown as mean ± SD (n = 3). Magnification: 10×, scale bar: 500 μm. *: *p* < 0.05, **: *p* < 0.01 and ***: *p* < 0.001 are compared with normoxia. ^†^: *p* < 0.05 and ^†††^: *p* < 0.001 are compared with the hypoxia group. ^ns^: denotes not significantly different from normoxia. ^NS^: denotes not significantly different from the hypoxia group.

The cell proliferative activity presented by the ki-67-positive cells was significantly lower in the hypoxia group than in the normoxia group (*p* < 0.001). However, the numbers of Ki-67-positive cells were markedly higher in the hypoxia group pretreated with rHDL, as well as Vit C, or HDL than in the hypoxia group (*p* ≤ 0.049) (Figure 7).

## 4. Discussion

The notable findings of this study are that rHDL reduces hypoxia-induced ROS production and RCF apoptosis, and that it facilitates cell proliferation of RCFs under hypoxic conditions.

Several studies have utilized CoCl_2_-induced hypoxia models, employing various biomolecular markers associated with different exposure times and concentrations across several cell types [33,34,35,36,37]. CoCl_2_ treatment upregulates hypoxia markers, including HIF-1α, HO-1, and BNIP3, in many cell types [38,39,40,41]. HIF-1α is recognized as the master regulator of the transcriptional cellular response to hypoxia [42]. Additionally, HO-1 is induced by various stimuli, including hypoxia and oxidative stress, serving as an adaptive cellular response that provides resistance to oxidative stress [43]. Under hypoxia, BNIP3, a member of the Bcl-2 family, undergoes upregulation and translocation to the mitochondria, disrupting mitochondrial membrane potential and triggering the release of cytochrome c, ultimately activating the caspase cascade [41]. In this study, the expressions of HIF-1α, HO-1, and BNIP3 were significantly higher in the hypoxia group than in the normoxia group (*p* ≤ 0.001), supporting previous studies’ results that CoCl_2_ increases expressions of the HIF-1α, HO-1, and BNIP3 [38,39,40,44].

In this study, the expressions of HIF-1α, HO-1, and BNIP3 were significantly attenuated by pretreatment with rHDL (*p* ≤ 0.028). Previous studies have reported that antioxidants, such as NAC or vitamin C, prevent the stabilization of hypoxia-induced HIF-1α and reduce the expression of hypoxia-induced HO-1 [45,46,47]. Furthermore, one study using human coronary artery endothelial cells demonstrated that rHDL augments the HIF-1α pathway via SR-BI (scavenger receptor class B type I) and modulation of the post-translational regulators of HIF-1α, which supports the findings of the current study [25]. Moreover, NAC mitigates the hypoxia-induced expression of BNIP3 [48]. The current study confirmed the findings of these previous studies by using vitamin C and NAC. Additionally, it newly demonstrated the ability of rHDL and HDL to attenuate the hypoxia-induced expressions of HIF-1α, HO-1, and BNIP3.

Cells experiencing hypoxic stress are known to produce increased intracellular ROS [49]. During hypoxia, ROS function as signaling agents, stabilizing the transcription factor HIF-1α and triggering various functional responses, including the activation of gene expressions [50]. CoCl_2_-induced hypoxia has been reported to increase the levels of ROS in several cell types such as PC12, periodontal cells and rat rotator cuff fibroblasts [27,51,52,53]. In the present study, intracellular ROS production was significantly higher in the hypoxia group than in the normoxia group, consistent with the findings of previous studies. Additionally, this study demonstrated a significant decrease in intracellular ROS production following pretreatment with rHDL or HDL and with well-known antioxidants, Vit C or NAC (*p* = 0.001). Antioxidant treatment reduced ROS and prevented HIF-1α stabilization under hypoxia [35,54]. Thus, this study confirms that pretreatment with rHDL or HDL, as well as with established antioxidants Vit C or NAC, effectively reduced the expression of HIF-1α.

Expressions of caspase-3 and of PARP-1 were significantly increased in the hypoxia group, as compared with the normoxia group in the present study. Caspase-3 is a recognized common executioner of both the intrinsic and extrinsic apoptosis pathways; PARP-1 also plays a role in the main pathways of apoptosis by stimulating the release of AIF [55,56]. Several studies have reported that hypoxia induces a significant increase in caspase-3 expression in several types of cells [57,58]. Several studies have reported that hypoxia induces a significant increase in PARP-1, and a co-modulating effect of HIF-1 and PARP-1 is involved in apoptotic cell death under hypoxia [59]. The present study aligns with previous research, confirming that hypoxia induced by CoCl_2_ promotes apoptosis and that caspase-3 and PARP-1 play a role in apoptotic process [27,60]. Furthermore, this study demonstrates a reduction in the elevated expressions of caspase-3 and PARP-1 through pretreatment with rHDL, or HDL, as well as Vit C or NAC. These findings elucidate the results of cell viability analyses, indicating that cell viability was significantly lower in the hypoxia group than in the normoxia group, while it was significantly higher in the hypoxia groups pretreated with NAC, Vitamin C, rHDL, or HDL compared to the hypoxia group.

In this study, the hypoxia group exhibited significantly increased expressions of VEGF and MMP-2 compared to the normoxia group. Hypoxia groups pretreatment with rHDL or HDL, as well as Vit C or NAC, resulted in significantly smaller increases in VEGF and MMP-2 expressions. ROS are essential mediators and modulators of the synthesis and activity of VEGF, a major angiogenic molecule [34,61], with HO-1 reported to be involved in angiogenesis by initiating the expression of VEGF [62,63]. This study supports the findings of previous studies in which hypoxia sequentially induced ROS, HO-1, and VEGF [63,64]. Antioxidants attenuate these hypoxia-induced increased productions of ROS and expressions of HO-1 and VEGF [27,65]. MMPs play roles in collagenolysis and elastolysis during periods of development, wound healing, and major inflammatory disease [66,67]. MMP-2 has been reported as expressed and activated during the healing process following acute supraspinatus tendon tear, during which MMP-2 is involved in the remodeling process [68]. MMP-2 degrades collagen types I, II, and III during matrix degradation [13,69,70,71,72,73]. The present study’s results suggest that hypoxia-induced degradation of the rotator cuff tendon matrix through the increased expression of MMP-2 is attenuated by pretreatment with rHDL or HDL, or with Vit C or NAC, which are well-known antioxidants.

Collagen types I and III play integral roles in tendon healing, with type I providing structural strength and integrity [74,75]. Conversely, collagen type III, which is more prevalent in early healing, contributes to matrix flexibility [74,75]. As healing progresses, collagen type III transitions to type I, forming a mature tendon structure [75]. This dynamic balance is crucial for optimal function, shifting from initial flexibility to increased strength [76]. In the current study, hypoxia decreased the production of collagen types I and III. However, rHDL, as well as NAC, Vit C, and HDL, each attenuated the production of collagens reduced by hypoxia. That finding indicated that rHDL preserves or stimulates tendon matrix production, suggesting the potential for rHDL in tendon healing or regeneration processes under hypoxic conditions.

Hypoxia can have dual effects on cell proliferation, depending on the hypoxia’s severity and the cell type. Hypoxia has been reported both to inhibit cell proliferation through activation of the p53 pathway and to stimulate proliferation via activation of the mTOR pathway [77,78]. The protein Ki67 is a marker for proliferating cells in active phases of the cell cycle, except for those cells in the G_0_ phase [79]. The present study found that the number of Ki67-positive cells was lower in the hypoxia group than in the normoxia group, while Ki67-positive cells increased in the hypoxia groups pretreated with rHDL or HDL, as well as in those pretreated with Vit C or NAC compared to the hypoxia group. These results suggest that rHDL can promote cell proliferation, an essential process for wound healing, under hypoxia.

A limitation of this study was that the studied hypoxia was induced with CoCl_2_, a mimicking agent, instead of using a hypoxic chamber. Additionally, the study did not determine whether CoCl_2_ could induce hypoxia in an animal model and whether rHDL would have novel effects on the prevention of hypoxia-induced apoptosis of the rotator cuff tendon in the animal model. Therefore, further studies should address these issues. While more research is needed to fully understand the mechanisms by which rHDL exerts anti-hypoxic effects, this study provides critical insight into the potential therapeutic benefits of rHDL in promoting tissue repair and regeneration in hypoxic conditions. These findings may also have broader implications for developing treatments for other diseases involving hypoxic damage and tissue degeneration.

## 5. Conclusions

In rotator cuff tendon fibroblasts under hypoxic conditions, rHDL enhances cell proliferation and collagen I and III production while reducing apoptosis, which can facilitate cuff tendon healing.

## Data Availability

Data are contained within the article and supplementary materials.

## Supplementary Materials

The following supporting information can be downloaded at: https://www.mdpi.com/article/10.3390/antiox13040497/s1, Figure S1: identification of rotator cuff fibroblasts; Figure S2: CoCl_2_-induced ROS production; Figure S3: CoCl_2_-induced HIF-1α, HO-1 and BNIP3 expressions; Figure S4: CoCl_2_-induced cell viability; Figure S5: CoCl_2_-induced apoptotic cell death; Figure S6: CoCl_2_-induced VEGF and MMP-2 expressions; Figure S7: CoCl_2_-induced collagen type I and collagen type III productions. Figure S8: DPPH radical scavenging activity of antioxidants.

## Abbreviations

BNIP3	Bcl-2/E1B-19kDa interacting protein 3
DAPI	4′, 6-diamidino-2-phenylindole
DPPH	2,2-diphenyl-1-picrylhydrazyl
HDL	high-density lipoprotein
HIF-1α	hypoxia-inducible factor-1α
HO-1	heme oxygenase-1
Hypo-HDLemia	hypo-high-density lipoproteinemia
MMP-2	matrix metalloproteinase-2
NAC	N-acetylcysteine
PARP-1	poly ADP-ribose polymerase-1
RCFs	rotator cuff fibroblasts
rHDL	reconstituted HDL
ROS	intracellular reactive oxygen species
VEGF	vascular endothelial growth factors
Vit C	vitamin C

## References

[1] Park H.B., Gwark J.Y., Im J.H., Jung J., Na J.B., Yoon C.H. (2018). Factors Associated with Atraumatic Posterosuperior Rotator Cuff Tears. J. Bone Jt. Surg. Am..

[2] Aagaard K.E., Bjornsson Hallgren H.C., Lunsjo K., Frobell R. (2022). No differences in histopathological degenerative changes found in acute, trauma-related rotator cuff tears compared with chronic, nontraumatic tears. Knee Surg. Sports Traumatol. Arthrosc..

[3] Karthikeyan S., Griffin D.R., Parsons N., Lawrence T.M., Modi C.S., Drew S.J., Smith C.D. (2015). Microvascular blood flow in normal and pathologic rotator cuffs. J. Shoulder Elbow Surg..

[4] Hegedus E.J., Cook C., Brennan M., Wyland D., Garrison J.C., Driesner D. (2010). Vascularity and tendon pathology in the rotator cuff: A review of literature and implications for rehabilitation and surgery. Br. J. Sports Med..

[5] Lohr J.F., Uhthoff H.K. (1990). The microvascular pattern of the supraspinatus tendon. Clin. Orthop. Relat. Res..

[6] Funakoshi T., Iwasaki N., Kamishima T., Nishida M., Ito Y., Kondo M., Minami A. (2010). In vivo visualization of vascular patterns of rotator cuff tears using contrast-enhanced ultrasound. Am. J. Sports Med..

[7] Yuan J., Murrell G.A., Wei A.Q., Wang M.X. (2002). Apoptosis in rotator cuff tendonopathy. J. Orthop. Res..

[8] Lakemeier S., Reichelt J.J., Patzer T., Fuchs-Winkelmann S., Paletta J.R., Schofer M.D. (2010). The association between retraction of the torn rotator cuff and increasing expression of hypoxia inducible factor 1alpha and vascular endothelial growth factor expression: An immunohistological study. BMC Musculoskelet. Disord..

[9] Lee H.J., Kim Y.S., Ok J.H., Song H.J. (2013). Apoptosis occurs throughout the diseased rotator cuff. Am. J. Sports Med..

[10] Pufe T., Petersen W.J., Mentlein R., Tillmann B.N. (2005). The role of vasculature and angiogenesis for the pathogenesis of degenerative tendons disease. Scand. J. Med. Sci. Sports.

[11] Fuhrmann D.C., Brune B. (2017). Mitochondrial composition and function under the control of hypoxia. Redox Biol..

[12] McBeath R., Edwards R.W., O’Hara B.J., Maltenfort M.G., Parks S.M., Steplewski A., Osterman A.L., Shapiro I.M. (2019). Tendinosis develops from age- and oxygen tension-dependent modulation of Rac1 activity. Aging Cell.

[13] Petersen W., Varoga D., Zantop T., Hassenpflug J., Mentlein R., Pufe T. (2004). Cyclic strain influences the expression of the vascular endothelial growth factor (VEGF) and the hypoxia inducible factor 1 alpha (HIF-1alpha) in tendon fibroblasts. J. Orthop. Res..

[14] Benson R.T., McDonnell S.M., Knowles H.J., Rees J.L., Carr A.J., Hulley P.A. (2010). Tendinopathy and tears of the rotator cuff are associated with hypoxia and apoptosis. J. Bone Jt. Surg. Br..

[15] Gerber H.P., Condorelli F., Park J., Ferrara N. (1997). Differential transcriptional regulation of the two vascular endothelial growth factor receptor genes. Flt-1, but not Flk-1/KDR, is up-regulated by hypoxia. J. Biol. Chem..

[16] Petersen W., Pufe T., Kurz B., Mentlein R., Tillmann B. (2002). Angiogenesis in fetal tendon development: Spatial and temporal expression of the angiogenic peptide vascular endothelial cell growth factor. Anat. Embryol..

[17] Lewis J.S., Raza S.A., Pilcher J., Heron C., Poloniecki J.D. (2009). The prevalence of neovascularity in patients clinically diagnosed with rotator cuff tendinopathy. BMC Musculoskelet. Disord..

[18] Abboud J.A., Kim J.S. (2010). The effect of hypercholesterolemia on rotator cuff disease. Clin. Orthop. Relat. Res..

[19] Park H.B., Gwark J.Y., Kwack B.H., Jung J. (2020). Hypo-High-Density Lipoproteinemia Is Associated with Preoperative Tear Size and With Postoperative Retear in Large to Massive Rotator Cuff Tears. Arthroscopy.

[20] Gordon D.J., Rifkind B.M. (1989). High-density lipoprotein--the clinical implications of recent studies. N. Engl. J. Med..

[21] Tan J.T., Ng M.K., Bursill C.A. (2015). The role of high-density lipoproteins in the regulation of angiogenesis. Cardiovasc. Res..

[22] Sumi M., Sata M., Miura S., Rye K.A., Toya N., Kanaoka Y., Yanaga K., Ohki T., Saku K., Nagai R. (2007). Reconstituted high-density lipoprotein stimulates differentiation of endothelial progenitor cells and enhances ischemia-induced angiogenesis. Arterioscler. Thromb. Vasc. Biol..

[23] Feng Y., van Eck M., Van Craeyveld E., Jacobs F., Carlier V., Van Linthout S., Erdel M., Tjwa M., De Geest B. (2009). Critical role of scavenger receptor-BI-expressing bone marrow-derived endothelial progenitor cells in the attenuation of allograft vasculopathy after human apo A-I transfer. Blood.

[24] Seetharam D., Mineo C., Gormley A.K., Gibson L.L., Vongpatanasin W., Chambliss K.L., Hahner L.D., Cummings M.L., Kitchens R.L., Marcel Y.L. (2006). High-density lipoprotein promotes endothelial cell migration and reendothelialization via scavenger receptor-B type I. Circ. Res..

[25] Tan J.T., Prosser H.C., Vanags L.Z., Monger S.A., Ng M.K., Bursill C.A. (2014). High-density lipoproteins augment hypoxia-induced angiogenesis via regulation of post-translational modulation of hypoxia-inducible factor 1alpha. FASEB J..

[26] Prosser H.C., Tan J.T., Dunn L.L., Patel S., Vanags L.Z., Bao S., Ng M.K., Bursill C.A. (2014). Multifunctional regulation of angiogenesis by high-density lipoproteins. Cardiovasc. Res..

[27] Kim R.J., An S.H., Gwark J.Y., Park H.B. (2021). Antioxidant effects on hypoxia-induced oxidative stress and apoptosis in rat rotator cuff fibroblasts. Eur. Cell Mater..

[28] Liang M., Cornell H.R., Zargar Baboldashti N., Thompson M.S., Carr A.J., Hulley P.A. (2012). Regulation of hypoxia-induced cell death in human tenocytes. Adv. Orthop..

[29] Vucic E., Rosenson R.S. (2011). Recombinant high-density lipoprotein formulations. Curr. Atheroscler. Rep..

[30] Cao Y.N., Xu L., Han Y.C., Wang Y.N., Liu G., Qi R. (2017). Recombinant high-density lipoproteins and their use in cardiovascular diseases. Drug Discov. Today.

[31] Goldberg M.A., Dunning S.P., Bunn H.F. (1988). Regulation of the erythropoietin gene: Evidence that the oxygen sensor is a heme protein. Science.

[32] Abdel-Rahman Mohamed A., Metwally M.M.M., Khalil S.R., Salem G.A., Ali H.A. (2019). Moringa oleifera extract attenuates the CoCl(2) induced hypoxia of rat’s brain: Expression pattern of HIF-1alpha, NF-kB, MAO and EPO. Biomed. Pharmacother..

[33] Borcar A., Menze M.A., Toner M., Hand S.C. (2013). Metabolic preconditioning of mammalian cells: Mimetic agents for hypoxia lack fidelity in promoting phosphorylation of pyruvate dehydrogenase. Cell Tissue Res..

[34] Huang Y.J., Nan G.X. (2019). Oxidative stress-induced angiogenesis. J. Clin. Neurosci..

[35] Lendahl U., Lee K.L., Yang H., Poellinger L. (2009). Generating specificity and diversity in the transcriptional response to hypoxia. Nat. Rev. Genet..

[36] Kim K.S., Rajagopal V., Gonsalves C., Johnson C., Kalra V.K. (2006). A novel role of hypoxia-inducible factor in cobalt chloride- and hypoxia-mediated expression of IL-8 chemokine in human endothelial cells. J. Immunol..

[37] Triantafyllou A., Liakos P., Tsakalof A., Georgatsou E., Simos G., Bonanou S. (2006). Cobalt induces hypoxia-inducible factor-1alpha (HIF-1alpha) in HeLa cells by an iron-independent, but ROS-, PI-3K- and MAPK-dependent mechanism. Free Radic. Res..

[38] Amersi F., Buelow R., Kato H., Ke B., Coito A.J., Shen X.D., Zhao D., Zaky J., Melinek J., Lassman C.R. (1999). Upregulation of heme oxygenase-1 protects genetically fat Zucker rat livers from ischemia/reperfusion injury. J. Clin. Investig..

[39] Taketani S., Kohno H., Yoshinaga T., Tokunaga R. (1988). Induction of heme oxygenase in rat hepatoma cells by exposure to heavy metals and hyperthermia. Biochem. Int..

[40] Wang G.L., Jiang B.H., Rue E.A., Semenza G.L. (1995). Hypoxia-inducible factor 1 is a basic-helix-loop-helix-PAS heterodimer regulated by cellular O2 tension. Proc. Natl. Acad. Sci. USA.

[41] Prabhakaran K., Li L., Zhang L., Borowitz J.L., Isom G.E. (2007). Upregulation of BNIP3 and translocation to mitochondria mediates cyanide-induced apoptosis in cortical cells. Neuroscience.

[42] Lu X., Kang Y. (2010). Hypoxia and hypoxia-inducible factors: Master regulators of metastasis. Clin. Cancer Res..

[43] Medina M.V., Sapochnik D., Garcia Sola M., Coso O. (2020). Regulation of the Expression of Heme Oxygenase-1: Signal Transduction, Gene Promoter Activation, and Beyond. Antioxid. Redox Signal.

[44] Azenshtein E., Meshel T., Shina S., Barak N., Keydar I., Ben-Baruch A. (2005). The angiogenic factors CXCL8 and VEGF in breast cancer: Regulation by an array of pro-malignancy factors. Cancer Lett..

[45] Gao P., Zhang H., Dinavahi R., Li F., Xiang Y., Raman V., Bhujwalla Z.M., Felsher D.W., Cheng L., Pevsner J. (2007). HIF-dependent antitumorigenic effect of antioxidants in vivo. Cancer Cell.

[46] Sceneay J., Liu M.C., Chen A., Wong C.S., Bowtell D.D., Moller A. (2013). The antioxidant N-acetylcysteine prevents HIF-1 stabilization under hypoxia in vitro but does not affect tumorigenesis in multiple breast cancer models in vivo. PLoS ONE.

[47] Motterlini R., Foresti R., Bassi R., Calabrese V., Clark J.E., Green C.J. (2000). Endothelial heme oxygenase-1 induction by hypoxia. Modulation by inducible nitric-oxide synthase and S-nitrosothiols. J. Biol. Chem..

[48] Yang L., Tan P., Zhou W., Zhu X., Cui Y., Zhu L., Feng X., Qi H., Zheng J., Gu P. (2012). N-acetylcysteine protects against hypoxia mimetic-induced autophagy by targeting the HIF-1alpha pathway in retinal ganglion cells. Cell Mol. Neurobiol..

[49] Aitio M.L. (2006). N-acetylcysteine -- passe-partout or much ado about nothing?. Br. J. Clin. Pharmacol..

[50] Guzy R.D., Schumacker P.T. (2006). Oxygen sensing by mitochondria at complex III: The paradox of increased reactive oxygen species during hypoxia. Exp. Physiol..

[51] Kotake-Nara E., Saida K. (2006). Endothelin-2/vasoactive intestinal contractor: Regulation of expression via reactive oxygen species induced by CoCl2, and Biological activities including neurite outgrowth in PC12 cells. ScientificWorldJournal.

[52] Zou W., Yan M., Xu W., Huo H., Sun L., Zheng Z., Liu X. (2001). Cobalt chloride induces PC12 cells apoptosis through reactive oxygen species and accompanied by AP-1 activation. J. Neurosci. Res..

[53] Song Z.C., Zhou W., Shu R., Ni J. (2012). Hypoxia induces apoptosis and autophagic cell death in human periodontal ligament cells through HIF-1alpha pathway. Cell Prolif..

[54] Lu H., Dalgard C.L., Mohyeldin A., McFate T., Tait A.S., Verma A. (2005). Reversible inactivation of HIF-1 prolyl hydroxylases allows cell metabolism to control basal HIF-1. J. Biol. Chem..

[55] McIlwain D.R., Berger T., Mak T.W. (2013). Caspase functions in cell death and disease. Cold Spring Harb. Perspect. Biol..

[56] Tewari M., Quan L.T., O’Rourke K., Desnoyers S., Zeng Z., Beidler D.R., Poirier G.G., Salvesen G.S., Dixit V.M. (1995). Yama/CPP32 beta, a mammalian homolog of CED-3, is a CrmA-inhibitable protease that cleaves the death substrate poly(ADP-ribose) polymerase. Cell.

[57] Deng C., Li J., Li L., Sun F., Xie J. (2019). Effects of hypoxia ischemia on caspase-3 expression and neuronal apoptosis in the brain of neonatal mice. Exp. Ther. Med..

[58] Khurana P., Ashraf Q.M., Mishra O.P., Delivoria-Papadopoulos M. (2002). Effect of hypoxia on caspase-3, -8, and -9 activity and expression in the cerebral cortex of newborn piglets. Neurochem. Res..

[59] Marti J.M., Garcia-Diaz A., Delgado-Bellido D., O’Valle F., Gonzalez-Flores A., Carlevaris O., Rodriguez-Vargas J.M., Ame J.C., Dantzer F., King G.L. (2021). Selective modulation by PARP-1 of HIF-1alpha-recruitment to chromatin during hypoxia is required for tumor adaptation to hypoxic conditions. Redox Biol..

[60] Mucci S., Isaja L., Rodriguez-Varela M.S., Ferriol-Laffouillere S.L., Marazita M., Videla-Richardson G.A., Sevlever G.E., Scassa M.E., Romorini L. (2022). Acute severe hypoxia induces apoptosis of human pluripotent stem cells by a HIF-1alpha and P53 independent mechanism. Sci. Rep..

[61] Kim Y.W., Byzova T.V. (2014). Oxidative stress in angiogenesis and vascular disease. Blood.

[62] Dulak J., Deshane J., Jozkowicz A., Agarwal A. (2008). Heme oxygenase-1 and carbon monoxide in vascular pathobiology: Focus on angiogenesis. Circulation.

[63] Dulak J., Loboda A., Jozkowicz A. (2008). Effect of heme oxygenase-1 on vascular function and disease. Curr. Opin. Lipidol..

[64] Sadaghianloo N., Yamamoto K., Bai H., Tsuneki M., Protack C.D., Hall M.R., Declemy S., Hassen-Khodja R., Madri J., Dardik A. (2017). Increased Oxidative Stress and Hypoxia Inducible Factor-1 Expression during Arteriovenous Fistula Maturation. Ann. Vasc. Surg..

[65] Chae H.S., Park H.J., Hwang H.R., Kwon A., Lim W.H., Yi W.J., Han D.H., Kim Y.H., Baek J.H. (2011). The effect of antioxidants on the production of pro-inflammatory cytokines and orthodontic tooth movement. Mol. Cells.

[66] Antonicelli F., Bellon G., Debelle L., Hornebeck W. (2007). Elastin-elastases and inflamm-aging. Curr. Top. Dev. Biol..

[67] Fields G.B. (2013). Interstitial collagen catabolism. J. Biol. Chem..

[68] Choi H.R., Kondo S., Hirose K., Ishiguro N., Hasegawa Y., Iwata H. (2002). Expression and enzymatic activity of MMP-2 during healing process of the acute supraspinatus tendon tear in rabbits. J. Orthop. Res..

[69] Aimes R.T., Quigley J.P. (1995). Matrix metalloproteinase-2 is an interstitial collagenase. Inhibitor-free enzyme catalyzes the cleavage of collagen fibrils and soluble native type I collagen generating the specific 3/4- and 1/4-length fragments. J. Biol. Chem..

[70] Kannus P. (2000). Structure of the tendon connective tissue. Scand. J. Med. Sci. Sports.

[71] Konttinen Y.T., Ceponis A., Takagi M., Ainola M., Sorsa T., Sutinen M., Salo T., Ma J., Santavirta S., Seiki M. (1998). New collagenolytic enzymes/cascade identified at the pannus-hard tissue junction in rheumatoid arthritis: Destruction from above. Matrix Biol..

[72] Patterson M.L., Atkinson S.J., Knauper V., Murphy G. (2001). Specific collagenolysis by gelatinase A, MMP-2, is determined by the hemopexin domain and not the fibronectin-like domain. FEBS Lett..

[73] Riley G. (2004). The pathogenesis of tendinopathy. A molecular perspective. Rheumatology.

[74] Kumagai J., Uhthoff H.K., Sarkar K., Murnaghan J.P. (1992). Collagen type III in rotator cuff tears: An immunohistochemical study. J. Shoulder Elbow Surg..

[75] Hirose K., Kondo S., Choi H.R., Mishima S., Iwata H., Ishiguro N. (2004). Spontaneous healing process of a supraspinatus tendon tear in rabbits. Arch. Orthop. Trauma. Surg..

[76] Singh D., Rai V., Agrawal D.K. (2023). Regulation of Collagen I and Collagen III in Tissue Injury and Regeneration. Cardiol. Cardiovasc. Med..

[77] Zhang C., Liu J., Wang J., Zhang T., Xu D., Hu W., Feng Z. (2021). The Interplay Between Tumor Suppressor p53 and Hypoxia Signaling Pathways in Cancer. Front. Cell Dev. Biol..

[78] Laplante M., Sabatini D.M. (2009). mTOR signaling at a glance. J. Cell Sci..

[79] Scholzen T., Gerdes J. (2000). The Ki-67 protein: From the known and the unknown. J. Cell Physiol..

