# Supplementary material for: Protective and Regenerative Effects of Reconstituted HDL on Human Rotator Cuff Fibroblasts under Hypoxia: An In Vitro Study
Source: Antioxidants (Basel). 2024 Apr 22;13(4):497. doi: 10.3390/antiox13040497 (PMC11047627; doi:10.3390/antiox13040497)
Supplement: Supplementary file 1 [file antioxidants-13-00497-s001.zip › antioxidants-2930368-supplementary.pdf]

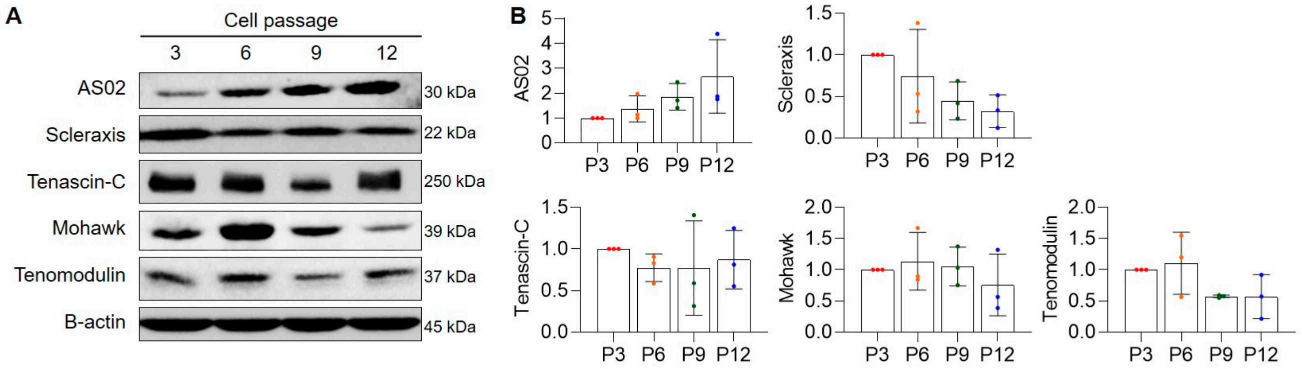

Supplement Figure S1. Western blot analysis for identifying rotator cuff fibroblasts (RCFs). (**A, B**) The studied cultured cells are characterized using specific cell markers, including AS02, which is a fibroblast marker, as well as scleraxis, tenascin-c, mohawk, and tenomodulin, which are tendon cell specific markers. The cultured cells expressed those cell markers, suggesting that the cultured cells are human RCFs. Data are shown as mean ± SD (n=3).

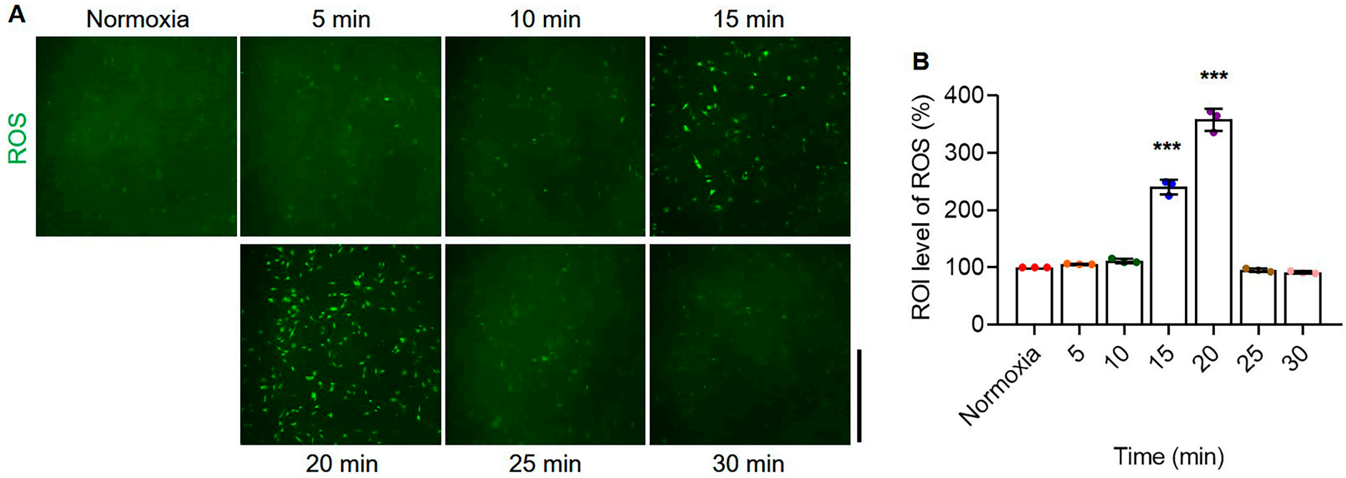

Supplement Figure S2. Fluorescence microscope analysis of RCFs exposed to $CoCl_2$. (**A**, **B**) Exposure of human RCFs to 1000 μM $CoCl_2$ induces a significant increase in the production of reactive oxygen species (ROS). Magnification: 4×. Scale bar: 2500 μm. Data are shown as mean ± SD (n=3). One data point represents the mean of technical replicates of an independent experiment. ***: $p < 0.001$ is compared with normoxia.

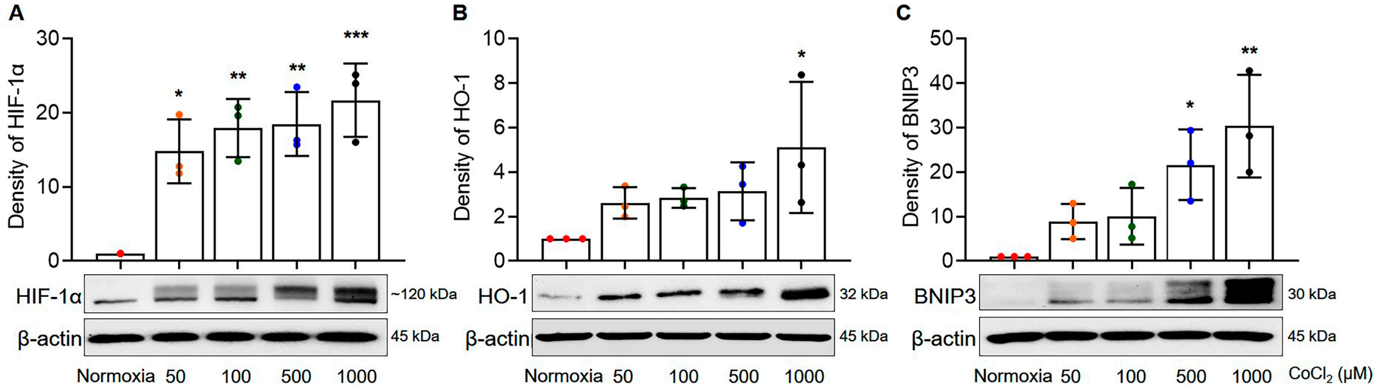

Supplement Figure S3. Western blot analysis of human RCFs exposed to $CoCl_2$. Exposure of human RCFs to $CoCl_2$ induces increased expressions of hypoxia-inducible factor-1α (HIF1-α), heme oxygenase-1 (HO-1), and Bcl-2/E1B-19kDa interacting protein 3 (BNIP3), which serve as markers of hypoxia. (**A**) HIF-1α expressions in all $CoCl_2$ study groups were significantly higher than in the normoxia group ($p \leq 0.011$). (**B**) HO-1 expression was significantly higher in the 1,000 μmol/L $CoCl_2$ study groups than in the normoxia group ($p = 0.044$). (**C**) BNIP3 expressions were significantly higher in the 500 and 1,000 μmol/L $CoCl_2$ study groups than in the normoxia group ($p \leq 0.033$). Data are shown as mean ± SD (n=3). *: $p < 0.05$, **: $p < 0.01$ and ***: $p < 0.001$ are compared with normoxia.

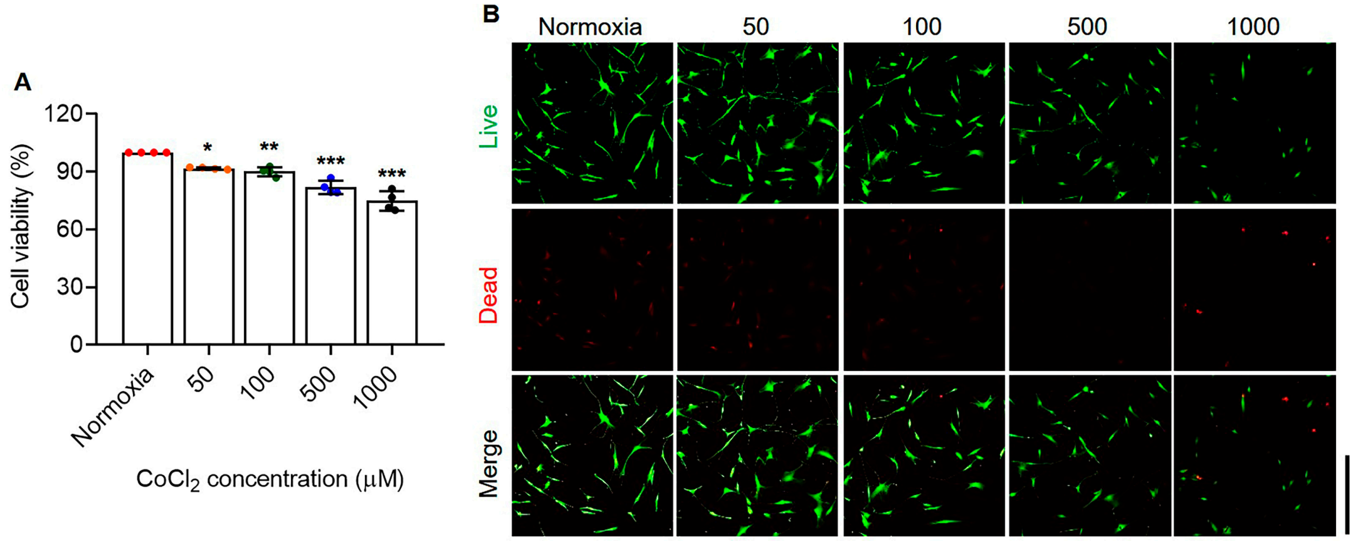

Supplement Figure S4. Effect of $CoCl_2$ on cell viability. (**A**) MTT test reveals that $CoCl_2$ significantly induces cell death ($p < 0.010$) (**B**) live and dead analyses reveal that $CoCl_2$ markedly increases cell death. Magnification: 10×, Scale bar: 500 μm, Data are shown as mean ± SD (n=4). One data point represents the mean of technical replicates of an independent experiment. *: $p < 0.05$, **: $p < 0.01$ and ***: $p < 0.001$ are compared with normoxia.

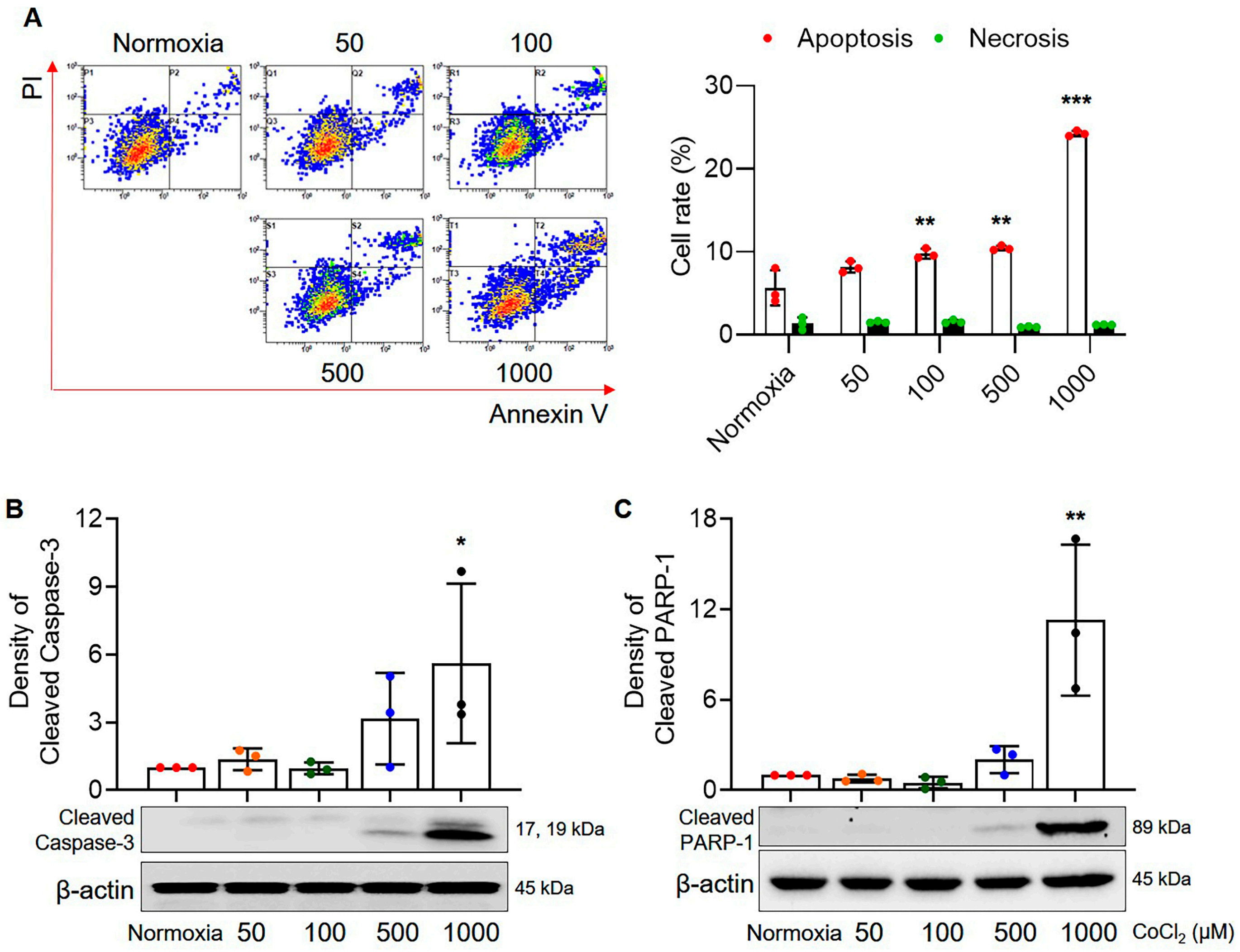

Supplement Figure S5. Effect of $CoCl_2$ on apoptosis. (A) Annexin V-PI double staining analyses show that the apoptosis rate was significantly higher in the 100, 500 and 1,000 μmol/L $CoCl_2$ study groups than in the normoxia group ($p \leq 0.005$). (B, C) Western blot analyses demonstrate that 1,000 μmol/L $CoCl_2$ induces an increase in the expression of cleaved caspase-3 ($p = 0.023$) and cleaved PARP-1 ($p = 0.002$). Data are shown as mean ± SD (n=3). *: $p < 0.05$, **: $p < 0.01$ and ***: $p < 0.001$ are compared with normoxia.

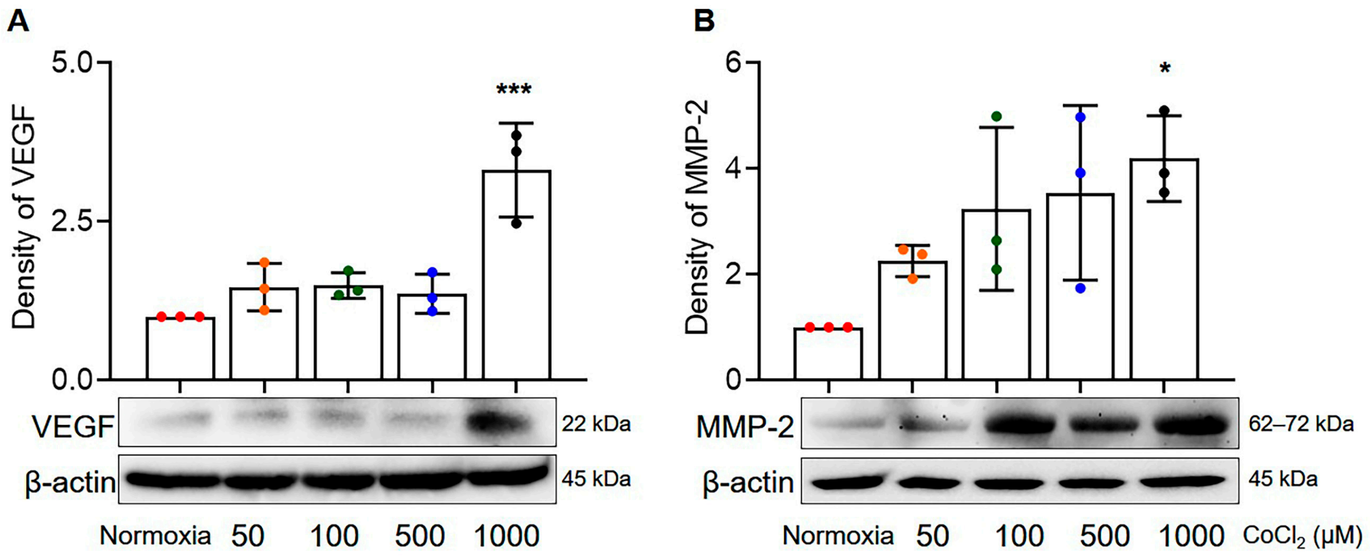

Supplement Figure S6. $CoCl_2$-induced changes in vascular endothelial growth factors (VEGF) and matrix metalloproteinase-2 (MMP-2) expressions. (**A**) VEGF expression was significantly higher in the 1,000 μmol/L $CoCl_2$ study group than in the normoxia group ($p < 0.001$). (**B**) MMP-2 expression was significantly higher in the 1,000 μmol/L $CoCl_2$ study group than in the normoxia group ($p < 0.030$). Data are shown as mean ± SD (n=3). *: $p < 0.05$ and ***: $p < 0.001$ are compared with normoxia.

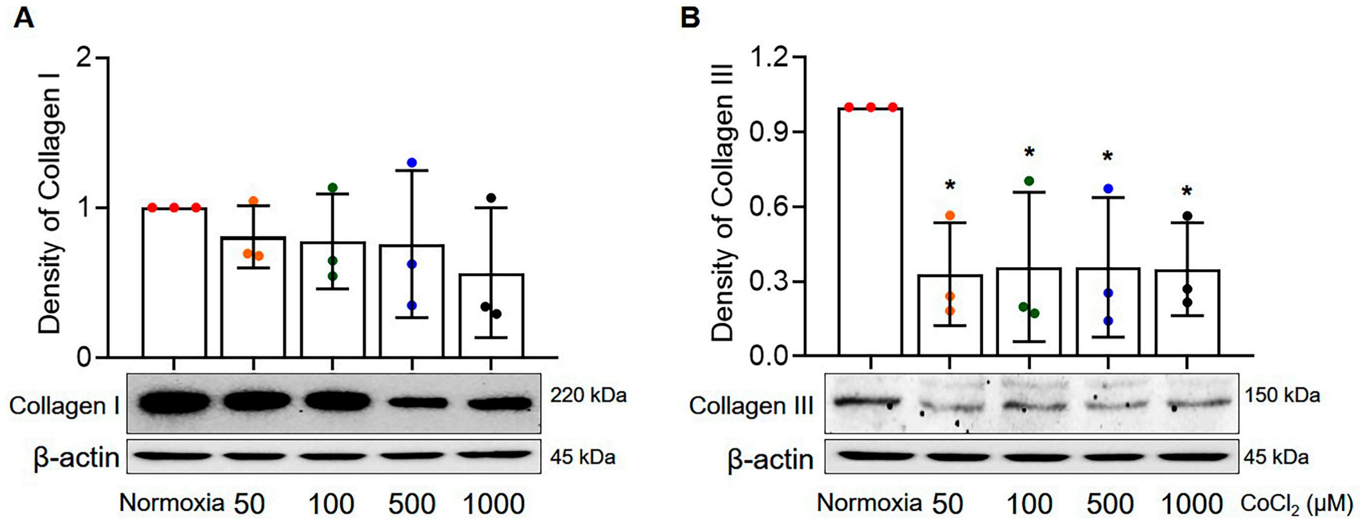

Supplement Figure S7. $CoCl_2$-induced changes in Collagen I and III expressions. $CoCl_2$ induces decreases in the expressions of Collagen I and III. (**A**) Collagen I expression was markedly lower in the 1,000 μmol/L $CoCl_2$ study group than in the normoxia group. (**B**) Collagen III expression in all $CoCl_2$ study groups were significantly lower than in the normoxia group ($p \leq 0.034$). Data are shown as mean ± SD (n=3). *: $p < 0.05$ is compared with normoxia.

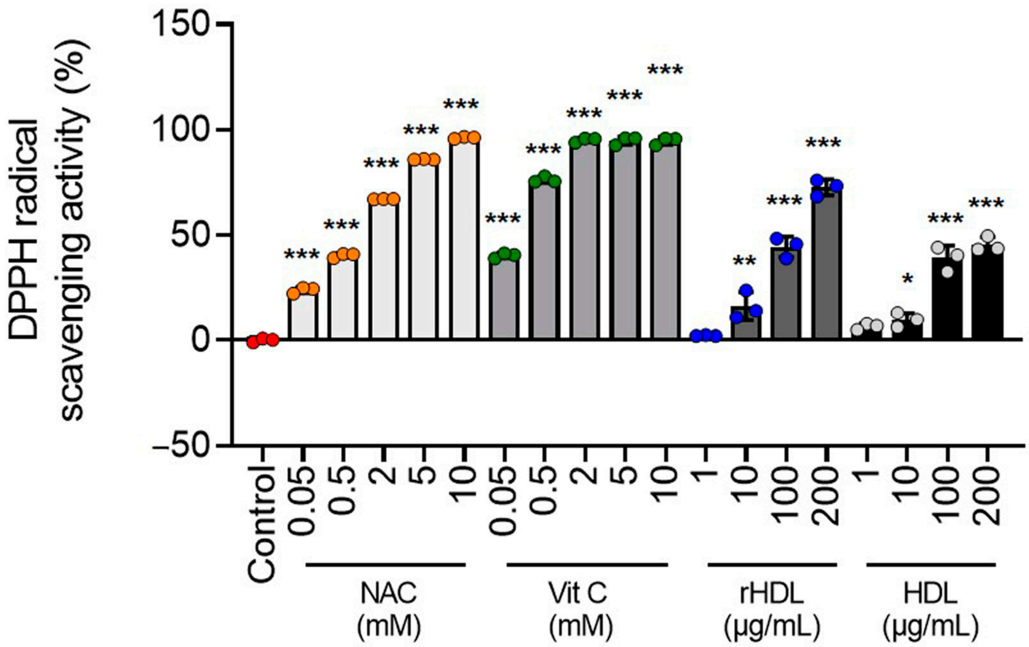

Supplement Figure S8. DPPH radical scavenging activity analysis. The analysis of 2,2-diphenyl-1-picrylhydrazyl (DPPH) radical scavenging activity reveals significant antioxidant capabilities in N-acetylcysteine (NAC), vitamin C (Vit C), reconstituted HDL (rHDL), and high-density lipoprotein (HDL) compared to their lowest concentrations. These results demonstrate that the studied rHDL exhibits significant oxidative radical scavenging capability, similar to NAC, Vit C, and HDL. Data are shown as mean ± SD (n=3). One data point represents the mean of technical replicates of an independent experiment. *: $p < 0.05$, **: $p < 0.01$ and ***: $p < 0.001$ are compared with the control.
